# *Gnaz* couples the circadian and dopaminergic system to G protein-mediated signaling in mouse photoreceptors

**DOI:** 10.1371/journal.pone.0187411

**Published:** 2017-10-31

**Authors:** Patrick Vancura, Shaima Abdelhadi, Erika Csicsely, Kenkichi Baba, Gianluca Tosini, P. Michael Iuvone, Rainer Spessert

**Affiliations:** 1 Institute of Functional and Clinical Anatomy, University Medical Center of the Johannes Gutenberg University, Mainz, Germany; 2 Neuroscience Institute and Department of Pharmacology and Toxicology, Morehouse School of Medicine, Atlanta, Georgia, United States of America; 3 Department of Ophthalmology, Emory University School of Medicine, Atlanta, Georgia, United States of America; McGill University, CANADA

## Abstract

The mammalian retina harbors a circadian clockwork that regulates vision and promotes healthiness of retinal neurons, mainly through directing the rhythmic release of the neurohormones dopamine—acting on dopamine D_4_ receptors—and melatonin—acting on MT_1_ and MT_2_ receptors. The gene *Gnaz*—a unique Gi/o subfamily member—was seen in the present study to be expressed in photoreceptors where its protein product Gα_z_ shows a daily rhythm in its subcellular localization. Apart from subcellular localization, *Gnaz* displays a daily rhythm in expression—with peak values at night—in preparations of the whole retina, microdissected photoreceptors and photoreceptor-related pinealocytes. In retina, *Gnaz* rhythmicity was observed to persist under constant darkness and to be abolished in retina deficient for *Clock* or dopamine D_4_ receptors. Furthermore, circadian regulation of *Gnaz* was disturbed in the *db*/*db* mouse, a model of diabetic retinopathy. The data of the present study suggest that *Gnaz* links the circadian clockwork—via dopamine acting on D_4_ receptors—to G protein-mediated signaling in intact but not diabetic retina.

## Introduction

The mammalian retina is known to harbor an intrinsic circadian clock system [1, 2] where circadian clocks are localized in various types of retinal neurons including horizontal cells, amacrine cells [3, 4] and photoreceptors [5–7]. The molecular clock enables the retina to adjust its physiology to adapt to daily changes in environmental demands. In particular, the retinal clock promotes adjustment of visual processing [8] that manifests in circadian changes in the retinal electrical responses to light, which can be measured using the ERG [9, 10]. Clock-dependent regulation of retinal physiology involves the neurohormones melatonin and dopamine [11, 12]. Both neurohormones play opposing roles in retinal adaptation. While melatonin by acting on MT_1_ and MT_2_ receptors promotes adaptation to darkness [13, 14] dopamine supports adaptation to light by acting on D_4_ receptors [15–18].

Heterotrimeric G proteins mediate the stimulation of G protein coupled receptors (GPCRs) to regulate a broad range of physiological functions in various tissues [19]. Each G protein consists of an α-subunit that binds and hydrolyzes GTP, as well as a β- and a γ-subunit. Sixteen types of α-subunits, five types of β-subunits and thirteen types of γ-subunits are known in humans. The sixteen α-subunits are encoded by a gene superfamily that can be subdivided into four different classes: *Gnai*, *Gnas*, *Gnaq* and *Gna12/13*. The *Gnai* class encompasses the four families *Gnai*, *Gnaz*, *Gnao* and *Gnat*. Based on their sequence similarities the *Gnaz* family—consisting of exclusively the gene *Gnaz*—and the *Gnat* family—involving the genes *Gnat1* and *Gnat2*—are considered α-transducins. However, *Gnaz* has no function in vision–and for this reason is referred to as *non-visual* α-transducin while *Gnat1* and *Gnat2* are involved in phototransduction and therefore are referred to as *visual* α-transducins.

The transcription of α-transducin display a 24-h rhythm in the rodent retina [20–22]. However, it is an open question whether the α-transducin investigated refers to the non-visual α-transducin *Gnaz* and/or the visual α-transducins *Gnat1* and/or *Gnat2*. The aim of the present study was to investigate (1) as to what extent expression of *Gnaz*, *Gnat1* and/or *Gnat2* is under daily regulation in retina, photoreceptors and photoreceptor-related pinealocytes, (2) depends on a circadian clock, (3) is regulated by the neurohormones melatonin and dopamine and (4) is disturbed in diabetic retinopathy.

## Material and methods

### Animals

Adult (age of 10–12 weeks) male and female mice (see below) not carrying *rd* mutations and, when indicated, rats (Sprague Dawley) were used in this study. With the exception of the mouse model for diabetic retinopathy (C57BL/6Jb db/+, C57BL/6Jb db/db), the mice used were melatonin-proficient (C3H/f^+/+^, C3H/f^+/+^Clock^+/+^, C3H/f^+/+^Clock^-/-^, C3H/f^+/+^MT1^+/+^, C3H/f^+/+^MT1^-/-^, C3H/f^+/+^Drd4^+/+^ and C3H/f^+/+^Drd4^-/-^). Genotyping was performed by PCR analysis of genomic DNA. C3H/f^+/+^Clock^+/+^ and C3H/f^+/+^Clock^-/-^ were generated by backcrossing Clock mice (strain name: B6.129S4-Clock^tm1.1Rep^/J) obtained from Jackson Laboratory (Bar Harbor, ME, USA) against C3H/f^+/+^ mice for ten generations. Diabetic (*db/db*) and non-diabetic (*db/+*) mice (strain name: BKS.Cq-*Dock7*^*m*^ +/+ *Lepr*^*db*^/J) were purchased from Jackson Laboratory. They were checked for body-weight and blood glucose level by tail vein sampling using Accu-Check Aviva reagent strips (Roche Diagnostics, Mannheim, Germany) at the age of 10 weeks. Diabetic mice displayed enhanced values of blood glucose (397 ± 14 mg/dl) and bodyweight (46 ± 3 g) as compared to non-diabetic mice (blood glucose level: 138 ± 4 mg/dl; bodyweight: 25 ± 1 g).

Animals were kept under light/dark 12:12 (LD) cycles for 3 weeks under standard laboratory conditions (illumination with 200 lux at cage level during the day and dim (< 5 lux) red light during the night, 20 ± 1°C, water and food ad libitum) and sacrificed at 3-h intervals over a period of 24 hours by decapitation following anesthesia with carbon dioxide. In order to determine putative clock-dependent regulation of genes, mice previously adapted to LD were housed in constant darkness (DD) for one cycle and sacrificed during the next cycle in DD. Animal experimentation was carried out in accordance with the National Institutes of Health Guide on the Care and Use of Laboratory Animals and the ARVO Statement for the Use of Animals in Ophthalmic Vision Research, and approved by the Institutional Animal Care and Use Committees of Morehouse School of Medicine, Emory University, and the European Communities Council Directive (86/609/EEC).

### Laser microdissection and pressure catapulting (LMPC)

To prepare the retinae for LMPC, the HOPE technique (DCS, Hamburg, Germany) was applied for fixation [23]. Photoreceptors were isolated from stained sections in a contact- and contamination-free manner by using the LMPC technique as described previously [6]. The purity grades of the preparations obtained were verified by using a specific gene marker of photoreceptors, namely *Nrl* as a marker for rods [24], and of inner retinal neurons, namely *Th* as a marker for amacrine cells [25]. In comparison to whole retina preparations, in photoreceptors collected by LMPC, the ratio of *Nrl* to *Th* was increased 84-fold.

### RNA extraction, reverse transcription (RT) and quantitative polymerase chain reaction (qPCR)

Using the RNeasy Micro Kit (Qiagen, Hilden, Germany) RNA was extracted from the tissue samples as described previously [26]. Subsequently first stranded cDNA was synthesized using the Verso cDNA Kit (Abgene, Hamburg, Germany), following the manufacturer’s instructions. Briefly, 4 μl RNA solution was reverse transcribed using anchored oligo-dT primers in a final volume of 20 μl. Following dilution of the obtained cDNA samples in RNase-free water (1:4) quantitative PCR was performed. Quantitative PCR was carried out in a total volume of 20 μL containing 10 μL ABsolute™ QPCR SYBR® Green Fluorescein Mix (Abgene), 0.2 μL of each primer (10 μM), 4.6 μL RNase-free water, and 5 μL sample. Primer sequences are listed in Table 1. PCR amplification and quantification were performed in duplicate using an i-Cycler (BioRad, Munich, Germany) according to the following protocol: denaturation for 30 seconds at 95°C, followed by 45 cycles of 5 seconds at 95°C and 30 seconds at 60°C. By using agarose gel electrophoresis, the generated amplicons for all genes under examination were shown to possess the predicted sizes (Table 1). To further confirm the specificity of the primer sets used—in particular of those for the genes *Gnaz*, *Gnat1* and *Gnat2—*sequencing of the generated amplicons was performed. According to the obtained sequences, the designed primer sets were verified as highly selective to their respective targets. The amount of mRNA in the samples was calculated from the measured threshold cycles (C_t_) using an internal standard curve with 10-fold serial dilutions (10^1^−10^8^ copies/μl). Expression levels of each transcript were normalized with respect to the amount of *Gapdh* mRNA and *18S* rRNA present.

**Table 1:** Primer sequences used for qPCR.

Gene	Accession Number	Primer Sequence 5′ to 3′	Length of PCR Product [bp]
*m18S*	NR_003278.3	Forward: CAACACGGGAAACCTCAC Reverse: TCGCTCCACCAACTAAGAAC	110
*mDrd4*	NM_007878.2	Forward: GTTGGACGCCTTTCTTCG Reverse: GTTGAGGGCACTGTTGAC	116
*mGapdh*	BC082592.1	Forward: CATCCCAGAGCTGAAC Reverse: TCAGATGCCTGCTTCAC	144
*mGna11*	NM_010301.3	Forward: GCATGACAGAGCCCTAGAG Reverse: ACAGGAGAGGAGCCTAGTG	106
*mGna12*	NM_010302.2	Forward: TGCAGGAGAACCTGAAAG Reverse: TGGTGTGGATTCGAGATG	149
*mGna13*	NM_010303.3	Forward: CCGTTGTACCACCACTTC Reverse: GAGCTGCTTCAGGTTGTC	102
*mGna14*	NM_008137.4	Forward: TCTGAACGACGGAAATGG Reverse: AAACAGGGCTTTGCTCTC	135
*mGna15*	NM_010304.3	Forward: TGAGCGAGTATGACCAGTG Reverse: CAGGATGTCCGTCTTGTTG	143
*mGnai1*	NM_010305.1	Forward: CCGCGTATATTCAGTGTC Reverse: CTGCACGTTCTTCGTATC	101
*mGnai2*	NM_008138.4	Forward: TGTTAGGTGCTGGAGAGTC Reverse: CTGGATGGTGTTGCTGTAG	125
*mGnai3*	NM_010306.3	Forward: AGCAGGTCCAGGGAATATC Reverse: CTCCACAATGCCTGTAGTC	138
*mGnal*	NM_010307.3	Forward: TGCTTCACAGTGGGAAATCG Reverse: GATGATACCGCTGGTAAAGTGG	114
*mGnao1*	NM_010308.3	Forward: GACGTGGTGAGTCGTATG Reverse: TACTCCCGAGATCGGTTG	116
*mGnaq*	NM_008139.5	Forward: GCCACAGCAGGATTGTTAAG Reverse: TTAAAGGGCAAGGGTGGAAG	120
*mGnas*	NM_010309.4	Forward: CAAGTTCCAGGTGGACAAAG Reverse: CCCGAATGACCATGTTGTAG	146
*mGnat1*	NM_008140.2	Forward: TTCGCCACAACGTCTATC Reverse: GTGTTAGGTCCATCGTAGTC	110
*mGnat2*	NM_008141.3	Forward: GCAGAGTTCCAGCTCAATG Reverse: CTCGATGATGCCTGTTGTC	129
*mGnaz*	NM_010311.3	Forward: GGTCTACATCCAACGTCAGTTC Reverse: TCTGTCACTGCGTCAAACAC	123
*mGnb1*	NM_008142.4	Forward: CTGTGGTGGCCTGGATAAC Reverse: CCGGCAACAGGACAGATAAC	112
*mGnb2*	NM_010312.4	Forward: GATTCCATGTGCCGACAG Reverse: GGTCAAAGAGGCGACAAG	121
*mGnb3*	NM_013530.1	Forward: CTGGCTGAGCTTGTGTCTG Reverse: CATCCTGCGAGGCACTTAC	142
*mGnb4*	NM_013531.4	Forward: TACTTCTGTGGCCTTCTC Reverse: CACACCTAAGCAGCTAAC	142
*mGnb5*	NM_010313.2	Forward: GCTATGCACACCAACTACC Reverse: GCTGTCCACTTTCCACATC	112
*mGngt1*	NM_010314.2	Forward: AGTCCTAGCTGTCCTGGAAATC Reverse: TGGCGCACGCCTTTAATAC	103
*mGngt2*	NM_023121.2	Forward: AAGGAGCTGTTGAGGATG Reverse: TCTTCTGGGATGCCTTTG	146
*mNrl*	NM_008736.3	Forward: GTGGAGGAACGGTCCAGATG Reverse: GAACTGGAGGGCTGGGTTAC	149
*mTh*	NM_009377.1	Forward: CAGCCCTACCAAGATCAAAC Reverse: GTACGGGTCAAACTTCACAG	129
*r18S*	NR_046237.1	Forward: GTTGGTGGAGCGATTTGTC Reverse: TCAATCTCGGTGGCTGAAC	136
*rDrd4*	NM_012944.1	Forward: TGGGCTATGTCAACAGTG Reverse: CATCAGCGGTTCTTTCAG	112
*rGapdh*	NM_017008.4	Forward: TGACTCTACCCACGGCAAG Reverse: CTGGAAGATGGTGATGGGTT	89
*rGnaz*	NM_013189.2	Forward: CCGAGTACAAGGGTCAGAAC Reverse: TCGGTGGCACAGGTAAAG	121

### Western blot analysis

For Western blot analysis, samples were loaded on 4–12% NuPAGE Novex Bis-Tris gels (Invitrogen, Carlsbad, CA, USA), separated and then blotted onto PVDF membrane (Westran S, Whatman Inc., Sanford, ME, USA). For immunodetection, membranes were blocked in 5% skim milk and incubated with rabbit polyclonal anti-Gα_z_ antibody (1:500; Santa Cruz Biotechnology, Santa Cruz, USA, sc-388) overnight at 4°C. Using an ECL detection system (GE Healthcare Amersham, Freiburg, Germany), the horseradish-peroxidase-conjugated secondary antibody (goat anti-rabbit-HRP 1:10.000; Sigma-Aldrich, St. Louis, MO, USA; A0545) was visualized. Monoclonal anti-β-actin HRP-coupled primary antibody (1:40.000; Sigma-Aldrich, St. Louis, MO, USA; A3854) was used to control for equal protein loading. Densitometry measurement was performed using Image Lab 4.1 (Bio-Rad Laboratories, Hercules, CA, USA).

### Fluorescence microscopy

Eyes were embedded in optimal cutting temperature (OCT) compound (Tissue-Tek; Sakura Finetek, Tokyo, Japan) and frozen in melting 2-methyl-butane (VWR, Radnor, PA, USA). Cryosections (10 μm) were treated with 0.1% Tween 20 in PBS, washed with PBS and then blocked with antibody diluent containing background reducing components (Dako, Capinteria, CA, USA) for 30 minutes at room temperature. Subsequently sections were incubated with primary antibodies (anti-Gα_z_ polyclonal antibody, 1:100, Santa Cruz Biotechnology, Santa Cruz, USA, sc-388; anti-Centrin3 polyclonal antibody, 1:100, kindly provided by Prof. Wolfrum, Institute of Zoology, Johannes Gutenberg University, Mainz, Germany) in antibody diluent overnight at 4°C. Following removal of the primary antibody, slides were washed with PBS and incubated with Alexa Fluor488 or Alexa Fluor568 conjugated donkey anti-mouse or donkey anti-rabbit secondary antibodies (Molecular Probes, Leiden, The Netherlands) for 1 hour in antibody diluent at room temperature. Cell nuclei were counterstained with DAPI (Thermo Fisher Scientific, Waltham, USA). Negative immunohistochemistry controls were performed in parallel by omission of primary antibodies. After they were washed, sections were cover slipped with fluorescent mounting medium (Dako, Capinteria, CA, USA). Stained retinal sections were examined by Axiophot microscope (Zeiss, Jena, Germany) and images were obtained with a digital imaging system (JVC, Yokohama, Japan).

### Statistical analysis

All data are expressed as the mean ± standard error of the mean (SEM) of four (qPCR and Western blot) independent experiments. Transcript levels were calculated relative to average expression of each dataset throughout 24 hours to plot temporal expression. Cosinor analysis was used to evaluate variations among the groups in the 24-h profile and to fit sine-wave curves to the circadian data to mathematically estimate the time of peaking gene expression (acrophase) and to assess the amplitude [27, 28]. The model can be expressed according to the equation: f(t) = A + B cos (2π (t + C) ⁄ T) with the f(t) indicating relative expression levels of target genes, t specifying the time of sampling (h), A representing the mean value of the cosine curve (mesor; midline estimating statistic of rhythm), B indicating the amplitude of the curve (half of the sinusoid) and C indicating the acrophase (point of time, when the function f(t) is maximum). T gives the time of the period, which was fixed at 24 hours for this experimental setting. Protein levels were calculated relative to actin immunoreactivity of each dataset throughout 24 hours to plot temporal expression. One-way ANOVA (one way analysis of variance) was used to evaluate variations among the groups in the 24-h profile. Significance of daily regulation was defined by showing a p < 0.05.

## Results

### *Gnaz* mRNA is under daily regulation in retina and pineal gland

**Fig 1:**
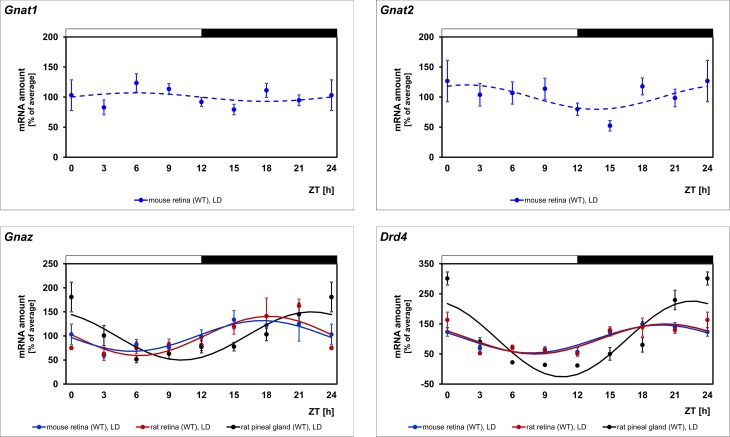
Daily profiling of the different types of α-transducins to be under daily regulation. Transcript levels of the non-visual α-transducin *Gnaz*, the visual α-transducins *Gnat1* and *Gnat2*, and the gene encoding the dopamine D_4_ receptor, *Drd4*, were measured using qPCR under LD in mouse retina (blue lines). *Gnaz* and *Drd4* mRNAs were also examined in rat retina (red lines) and rat pineal gland (black lines). The mRNA levels are plotted as a function of ZT and the lines represent the periodic sinusoidal functions determined by cosinor analysis (solid and broken line for p < 0.05 and p > 0.05 in cosinor analysis). Data represent a percentage of the average value of the transcript amount during the 24-h period. Statistical analysis of transcriptional profiling is provided in Table 2. The value of ZT0 is plotted twice at both ZT0 and ZT24. The solid bars indicate the dark period. Each value represents mean ± SEM (n = 4; each n represents two retinae and a pineal gland of one animal). Note that the mRNA levels of exclusively *Gnaz* and *Drd4* exhibit significant variations in all applied settings.

To gain insight into the daily regulation of G protein-dependent signal transduction in the mouse retina, transcript levels of different Gα subunits were determined as a function of time-of-day. Among the Gα subunits tested, exclusively the *non-visual* α-transducin gene *Gnaz* displayed a daily rhythm (Fig 1, blue lines; for statistical analysis, see Table 2). Neither of the *visual* α-transducins, *Gnat1* and *Gnat2* (Fig 1, blue lines; for statistical analysis, see Table 2), nor any of the other Gα subunits tested (*Gna11*, *Gna12*, *Gna13*, *Gnal4*, *Gnal5*, *Gnai1*, *Gnai2*, *Gnai3*, *Gnal*, *Gnao1*, *Gnaq*, *Gnas*) displayed a 24-h rhythm (data not shown). Similarly, rhythmic expression of Gβ subunits (*Gnb1*, *Gnb2*, *Gnb3*, *Gnb4*, *Gnb5*), and Gγ subunits (*Gngt1*, *Gngt2*) was not observed (data not shown).

**Table 2:** Statistical analysis of transcriptional profiling illustrated in Fig 1 and Figs 4–6.

Source of transcriptomes	*Gnaz*			*Drd4*			see Figure
	p-value	acrophase (h)	amplitude (%)	p-value	acrophase (h)	amplitude (%)	
**mouse retina (C3H/f**^**+/+**^ **(rd**^**++**^**)); WT; LD**	< 0.05	16.9	31.9	< 0.05	19.6	46.5	Fig 1, Fig 4
**rat retina (Sprague-Dawley); LD**	< 0.05	18.2	40.8	< 0.05	20.1	49.9	Fig 1
**rat pineal gland (Sprague-Dawley); LD**	< 0.05	21.5	50.8	< 0.05	22.6	126.2	Fig 1
**mouse photoreceptors (C3H/f**^**+/+**^ **(rd**^**++**^**)); WT; LD**	< 0.05	19.9	37.5	< 0.05	19.7	49.0	Fig 4
**mouse retina (C3H/f**^**+/+**^ **(rd**^**++**^**)); WT; DD**	< 0.05	18.8	29.2	< 0.05	17.9	29.1	Fig 4
**mouse retina (C3H/f**^**+/+**^ **(rd**^**++**^**)); Clock**^**+/+**^**; LD**	< 0.05	16.9	31.9	< 0.05	19.6	46.5	Fig 5
**mouse retina (C3H/f**^**+/+**^ **(rd**^**++**^**)); Clock**^**-/-**^**; LD**	> 0.05	-	-	> 0.05	-	-	Fig 5
**mouse retina (C3H/f**^**+/+**^ **(rd**^**++**^**)); MT1**^**+/+**^**-; LD**	< 0.05	18.2	28.2	< 0.05	19.1	51.8	Fig 5
**mouse retina (C3H/f**^**+/+**^ **(rd**^**++**^**)); MT1**^**-/-**^**; LD**	< 0.05	16.7	33.1	< 0.05	19.0	62.5	Fig 5
**mouse retina (C3H/f**^**+/+**^ **(rd**^**++**^**)); Drd4**^**+/+**^**; LD**	< 0.05	17.9	33.8	< 0.05	18.7	47.3	Fig 5
**mouse retina (C3H/f**^**+/+**^ **(rd**^**++**^**)); Drd4**^**-/-**^**; LD**	> 0.05	-	-	n. d.	-	-	Fig 5
**mouse retina (C57BL/6Jb); db/+; LD**	< 0.05	18.6	11.1	< 0.05	18.5	20.1	Fig 6
**mouse retina (C57BL/6Jb); db/db; LD**	> 0.05	-	-	> 0.05	-	-	Fig 6

*Gnaz* rhythmicity displayed peak expression in darkness and was similar in retina of mouse and rat (Fig 1, blue versus red lines; for statistical analysis, see Table 2). It also occurred in rat pineal gland, an organ that is phylogenetically related to the retina and is controlled by the body’s master clock in the suprachiasmatic nucleus [29] (Fig 1, black lines; for statistical analysis, see Table 2). This suggests that daily regulation of *Gnaz* is phylogenetically conserved and may in retina and pineal gland be promoted by different clocks.

### Daily regulation of Gα_z_ protein amount

**Fig 2:**
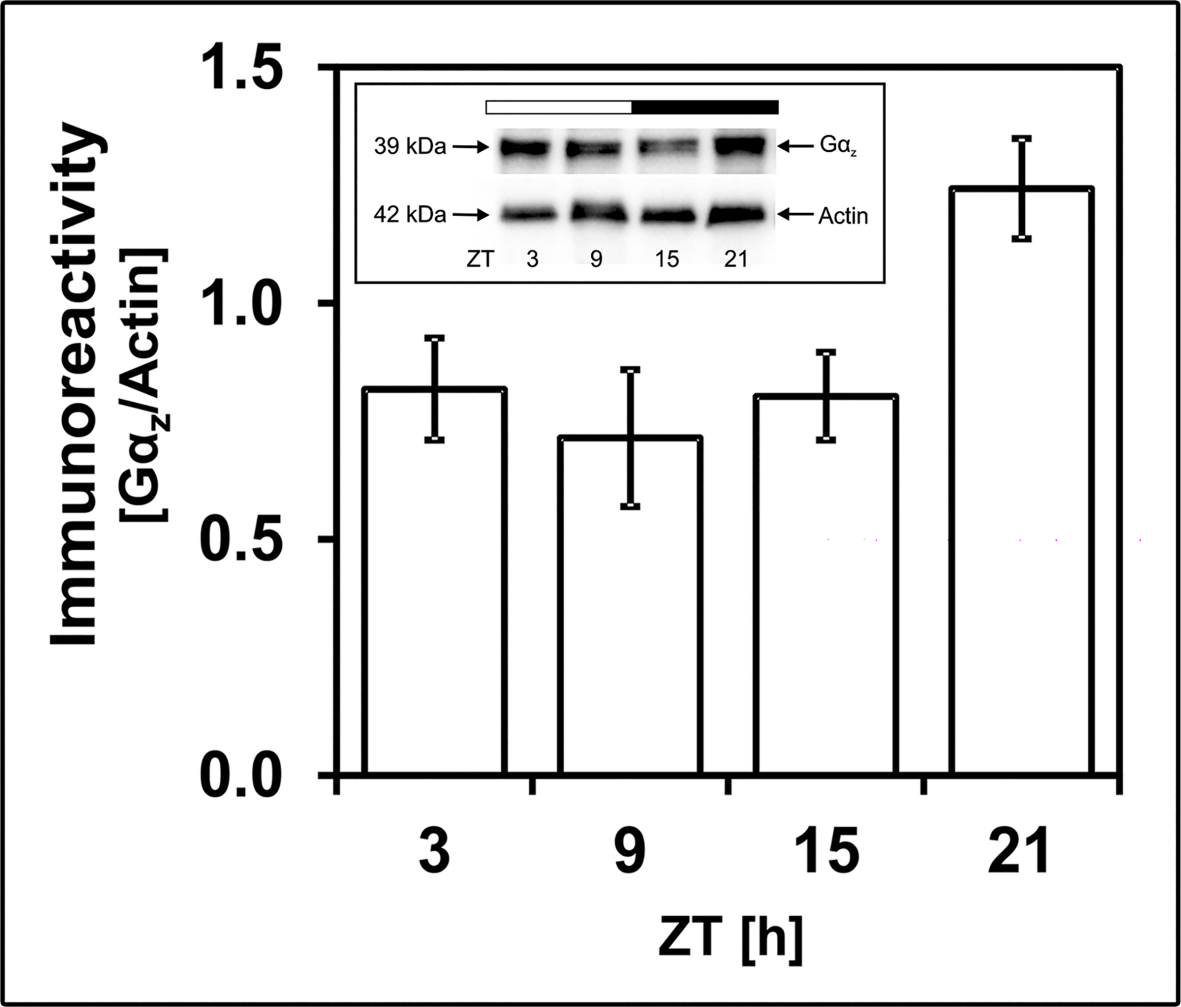
24-h profiling of Gα_z_ immunoreactivity. The figure shows a representative Western blot with Gα_z_ immunostaining at 39 kDa and β-actin staining as a loading control. The diagram displays the quantification of Gα_z_ immunoreactivity in relation to the corresponding β-actin signal. Data were obtained using densitometric measurement. Each value represents mean ± SEM (n = 4; each n represents two animals (four retinae)). Note that Gα_z_ immunoreactivity tends to exhibit daily changes with peak expression around ZT21 (p = 0.062 in one-way ANOVA). The solid bar indicates the dark period.

To investigate whether the observed variations in *Gnaz* mRNA result in corresponding variations in protein amount, Gα_z_ immunoreactivity was compared at different ZTs in Western blot analysis by using an antibody that recognizes a band of ~ 39 kDa (Fig 2), a molecular mass in the range of the predicted size from the *Gnaz* gene (355 amino acids). The intensity of Gα_z_ immunostaining tended to display a daily change (p = 0.062 in one-way ANOVA) with peak values around ZT21 (Fig 2). The temporal gap between the peaks in *Gnaz* mRNA (ZT16.9) and Gα_z_ protein (ZT21) may reflect the time necessary to translate the transcript into protein. Thus, this observation suggests that daily regulation of *Gnaz* mRNA amount causes a corresponding rhythm of Gα_z_ protein.

### Daily regulation of Gα_z_ protein localization

**Fig 3:**
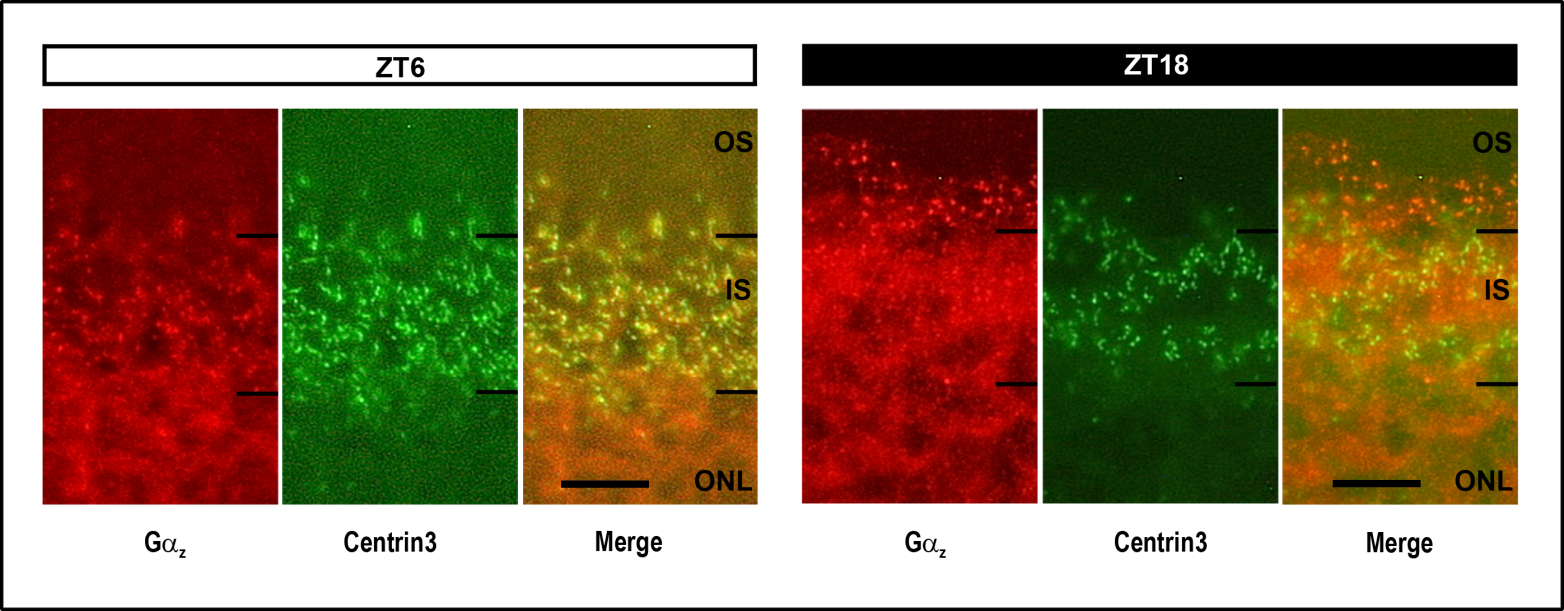
Daily translocation of Gα_z_ immunoreactivity. Micrographs of coronal sections of the retina, labelled for Gα_z_ (red) and centrin3 (green), a marker of the connecting cilium and the photoreceptor inner segment (IS). The representative immunofluorescent image shows that Gα_z_ protein is abundant in photoreceptors where its subcellular localization is under daily regulation. Gα_z_ immunoreactivity mainly occurs in the inner segment (IS) at ZT6 and in the outer segment (OS) at ZT18. The solid bars indicate the dark period. ONL, outer nuclear layer. Scale bar, 10 μm.

Localization of Gα_z_ protein was investigated in fluorescence microscopy (Fig 3) by conducting double labeling analysis for Gα_z_ and centrin3, a marker of the connecting cilium and the inner segment of photoreceptors [30]. Gα_z_ immunoreactivity mainly occurred in photoreceptors where its subcellular localization was seen to vary between day and night. This follows from the observation that Gα_z_ staining mainly arose in the connecting cilium/inner segment at ZT6 and was most dense in the outer segment at ZT18 (Fig 3). This supports a concept in which subcellular localization of Gα_z_ protein might exhibit a lighting condition-dependent transport from the connecting cilium/the inner segment to the outer segment.

### Daily regulation of *Gnaz* mRNA amount in photoreceptor cells

**Fig 4:**
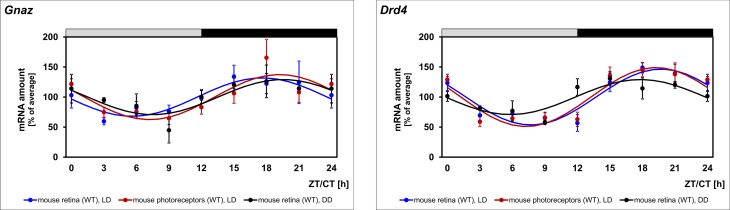
Daily profiling of *Gnaz* mRNA in photoreceptors and constant darkness. Transcript levels of *Gnaz* and *Drd4* were monitored under LD in mouse retina (blue lines) and mouse photoreceptors (red lines), as well as in mouse retina under DD (black lines) using qPCR. The mRNA levels are plotted as a function of ZT and circadian time (CT). The lines represent the periodic sinusoidal functions determined by cosinor analysis. Data represent a percentage of the average value of the transcript amount during the 24-h period. Statistical analysis of transcriptional profiling is provided in *Table 2*. Note that *Gnaz* mRNA rhythmicity is also evident in photoreceptors and persists in constant darkness. The value of ZT0 is plotted twice at both ZT0 and ZT24. The solid bars indicate the dark period. Each value represents mean ± SEM (n = 4; each n represents one animal (two retinae) for whole retina preparations and two animals (four retinae) for photoreceptor preparations).

The expression of *Gnaz* in photoreceptors raises the question whether rhythmicity of *Gnaz* mRNA arises from this cell type. To address this question, daily profiling of *Gnaz* mRNA was performed in photoreceptors enriched by using the LMPC technique. *Gnaz* transcript amount was seen to display a daily rhythm (Fig 4, red lines; for statistical analysis, see Table 2) with a 24-h profile resembling that obtained from preparations of the whole retina (Fig 4, blue lines; for statistical analysis, see Table 2). Therefore, daily changes in retinal *Gnaz* mRNA amount may partly or fully derive from photoreceptors.

### *Gnaz* expression depends on a circadian regulator

**Fig 5:**
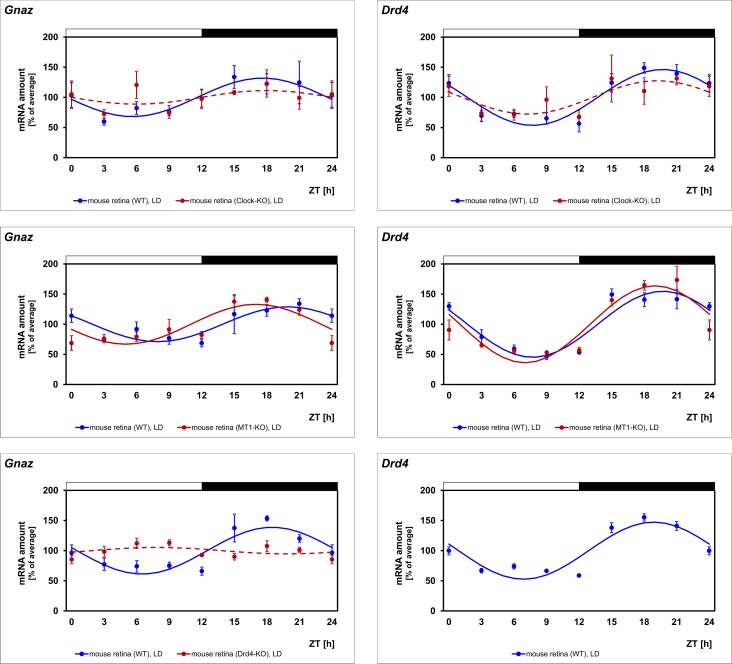
Daily profiling of *Gnaz* mRNA in mice deficient for *Clock*, *MT1* or *Drd4*. Transcript levels of *Gnaz* and *Drd4* were recorded in WT mice (blue lines) versus mice deficient (red lines) for *Clock* (first row), melatonin receptor type 1 (second row) or dopamine D_4_ receptor (third row) in preparations of the whole retina under LD using qPCR. The mRNA levels are plotted as a function of ZT. The lines represent the periodic sinusoidal functions determined by cosinor analysis (solid and broken line for p < 0.05 and p > 0.05). Data represent a percentage of the average value of the transcript amount during the 24-h period. Statistical analysis of transcriptional profiling is provided in Table 2. Note that expression of *Gnaz* is arrhythmic in mice deficient for *Clock* or dopamine D_4_ receptors and tends to be phase-advanced in mice deficient for *MT1*. The value of ZT0 is plotted twice at both ZT0 and ZT24. The solid bars indicate the dark period. Each value represents mean ± SEM (n = 4; each n represents one animal (two retinae)). *Drd4* mRNA was not detectable in *Drd4* deficient retinae.

24-h regulation of a gene may be promoted by a true circadian clock or light/dark-transitions. To test circadian regulation of *Gnaz*, 24-h profiling of transcript amount was conducted in mice adapted to DD (Fig 4, black lines; for statistical analysis, see Table 2). Consistent with clock-dependent regulation of *Gnaz* expression, the daily rhythm of *Gnaz* transcript persisted under DD. Furthermore, *Gnaz* was not rhythmically expressed in *Clock* deficient mice (Fig 5, first row, blue versus red lines; for statistical analysis, see Table 2). This supports the concept that *Gnaz* rhythmicity is driven by a retinal clock that requires *Clock* for its functionality [31] and not by the master clock in the suprachiasmatic nucleus (SCN), which does not require *Clock* for its functionality [32–34].

### Circadian regulation of *Gnaz* requires dopamine D_4_ receptors

In order to evaluate the contribution of melatonin and dopamine to daily regulation of *Gnaz*, 24-h profiling of the gene was performed in mice deficient for *MT1* (Fig 5, second row, blue versus red lines; for statistical analysis, see Table 2) or *Drd4* (Fig 5, third row, blue versus red lines; for statistical analysis, see Table 2). The daily rhythm of *Gnaz* was seen to persist in *MT1* deficient mice but peak expression appeared to be slightly phase-advanced. This observation suggests that melatonin signaling via MT_1_ receptors does not drive rhythmicity of *Gnaz* but might influence its phasing. More importantly, daily regulation of *Gnaz* was absent in *Drd4* deficient mice. This suggests that dopamine and D_4_ receptors play a role in driving circadian changes in *Gnaz* expression. In mouse retina dopamine release and D_4_ receptor stimulation occurs in a circadian manner [12]. Therefore, this finding supports the concept that *Gnaz* expression depends on a clock-driven dopamine signal.

### Expression of *Gnaz* is arrhythmic in diabetic retina

**Fig 6:**
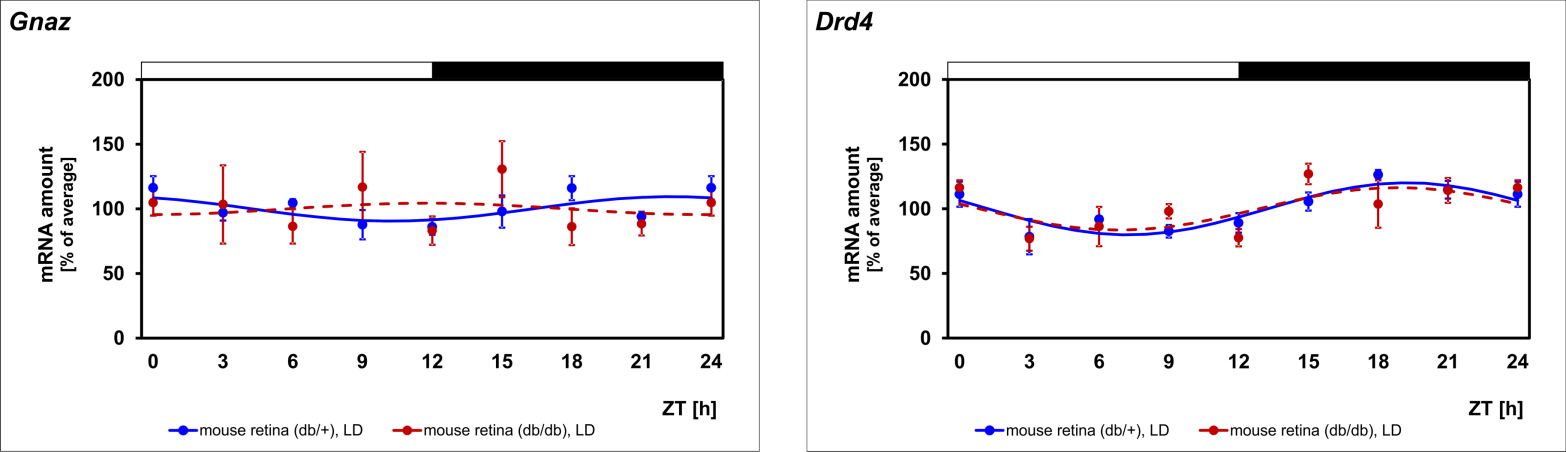
Daily profiling of *Gnaz* mRNA in diabetic retina. Transcript levels of *Gnaz* and *Drd4* were recorded in non-diabetic (*db/+*) mice (blue lines) versus diabetic (*db/db*) mice (red lines) in preparations of the whole retina under LD using qPCR. The mRNA levels are plotted as a function of ZT. The lines represent the periodic sinusoidal functions determined by cosinor analysis (solid and broken line for p < 0.05 and p > 0.05). Data represent a percentage of the average value of the transcript amount during the 24-h period. Statistical analysis of transcriptional profiling is provided in Table 2. Note that *Gnaz* expression is arrhythmic in diabetic mice. The value of ZT0 is plotted twice at both ZT0 and ZT24. The solid bars indicate the dark period. Each value represents mean ± SEM (n = 4; each n represents one animal (two retinae)).

To test the assumption that diabetic retinopathy impairs circadian control of *Gnaz*, the *db/db* mouse, a model of Type II diabetes [35] was used. The non-diabetic phenotype (*db/+*) was seen to display a daily rhythm in *Gnaz* mRNA but with a lower amplitude than that observed in previous experiments (Fig 6, blue lines; for statistical analysis, see Table 2). This may be due to a different genetic background of the *db/db* mice (C57BL/6Jb) and the other mouse strains, which were on a C3H background. Different to the non-diabetic phenotype (*db/+*), *Gnaz* expression was arrhythmic in diabetic (*db/db*) mice (Fig 6, blue versus red lines; for statistical analysis, see Table 2). Therefore, circadian regulation of *Gnaz* appears to be disturbed in diabetic retinopathy.

### Daily profiling of the clock-dependent gene *Drd4* confirms the validity of the experimental system

To test the validity of the experimental system and the obtained results, the clock-driven gene *Drd4* was monitored in the same transcriptomes as those utilized for *Gnaz* mRNA determination. Consistent with the validity of the results obtained for *Gnaz*, *Drd4* expression was observed to be rhythmic in retina of mouse and rat and pineal gland (Fig 1, for statistical analysis, see Table 2). As expected for a gene under circadian regulation, *Drd4* rhythmicity persisted under DD (Fig 4, black lines; for statistical analysis, see Table 2) and was damped in mice deficient for *Clock* (Fig 5, first row, blue versus red lines; for statistical analysis, see Table 2). Beyond what was previously known, *Drd4* rhythmicity was seen in the present study to persist in mice deficient for *MT1* (Fig 5, second row, blue versus red lines; for statistical analysis, see Table 2) and, according to statistical analysis, not in diabetic (*db/db*) mice (Table 2; Fig 6, blue versus red lines).

## Discussion

The gene *Gnaz* encodes Gα_z_, a unique Gi/o subfamily member, whose tissue distribution is quite restricted to primarily neuronal and endocrine tissue [36], including retina [37–39]. The findings of the present study extend previous knowledge on *Gnaz*/Gα_z_ distribution [37, 40–42] by showing that it is highly expressed in photoreceptors and pinealocytes—both cell types originating phylogenetically and ontogenetically from a common ancestral cell type [29].

*Gnaz* mRNA—but not *Gnat1* or *Gnat2 mRNA—*displays a daily rhythm in retina. This finding and large sequence similarities of *Gnaz* with *Gnat1* and *Gnat2* (44% and 43% respectively), suggest that the earlier reported daily change in α-transducin mRNA [20–22] relies on *Gnaz* but not on the visual types of α-transducin. This assumption is furthermore supported by the fact that in transcriptomes of the murine retina, *Gnaz* displays a higher day/night change than *Gnat2* and *Gnat1* does not undergo daily regulation at all [8]. Additionally the oligodeoxynucleotide probes used previously to detect α-transducin [20] are highly complementary with all types of α-transducin (*Gnaz*: 82%, *Gnat1*: 93%, *Gnat2*: 79%).

In the context of the functional significance of the rhythmic expression of the *Gnaz* gene, it is noteworthy that daily rhythmicity is also evident at the level of Gα_z_ protein. The daily profile in G*naz* mRNA resembles that in Gα_z_ protein. This suggests that daily regulation of *Gnaz* mRNA evokes a corresponding rhythm in Gα_z_ protein. Since Gα_z_ protein expression predominates in photoreceptors (this study), but may also be abundant in the inner retina [37], *Gnaz* expression might be under daily/circadian regulation not only in photoreceptors but also in inner retinal neurons. This assumption is consistent with the observation that the acrophase of *Gnaz* expression tends to differ in microdissected photoreceptors (ZT19.9) and preparations of the whole retina (ZT16.9). Furthermore, *Gnaz*/Gα_z_ is under daily regulation not only in respect to its expression but also in its subcellular localization within photoreceptors. This follows from the finding that Gα_z_ immunostaining was most dense in the connecting cilium/inner segment at ZT6 but in the outer segment at ZT18. Since Gα_z_ de-novo formation should occur in the inner segment, the temporary localization of Gα_z_ protein in the outer segment suggests that newly synthesized Gα_z_ is transported from the inner to the outer segment of photoreceptors.

Regulation of *Gnaz* expression was observed to be driven by a circadian clock. This is evident from the present observation that *Gnaz* rhythmicity persists under constant darkness and therefore does not require light/dark transitions. Circadian control of *Gnaz* appears to be driven by the retinal circadian clock system and not by the master clock in the SCN. This follows from the finding that *Gnaz* rhythmicity was not observed in *Clock-*deficient mice, in which circadian rhythms persist in the SCN due to the CLOCK homologue NPAS2 [32–34].

Rhythmicity of *Gnaz* was also evident in the pineal gland, a neuroendocrine transducer of the circadian system [43]. The pineal gland in general and its gene expression in particular are mainly under the control of the master clock in the SCN [44]. This suggests that *Gnaz* expression is circadian in both, retina and pineal gland, but in the retina depends on retinal clocks and in the pineal gland on the master clock in the SCN. The coincidence of *Gnaz* rhythmicity in mammalian photoreceptors and pinealocytes suggests that circadian regulation of *Gnaz* is evolutionary conserved. In both tissues, rhythmicity of *Gnaz* may depend on the clock-driven release of neurotransmitters. Thus circadian regulation of *Gnaz* may be mediated by dopamine in the retina (see below) and by noradrenaline in the pineal gland–the intra-pineal release of the latter neurotransmitter is known to be under the control of the SCN [45].

In murine retina, 24-h regulation of *Gnaz* appears to be mediated by dopamine signaling via D_4_ receptors. This follows from the present observation that the lack of functional D_4_ receptors prevents daily periodicity of *Gnaz*. Since photoreceptors combine daily rhythmicity of *Gnaz* with the occurrence of D_4_ receptors [15], dopaminergic regulation of Gα_z_ may mainly occur in photoreceptors. D_4_ receptor-mediated regulation of photoreceptor function appears to depend on the clock-driven [46, 47] release of dopamine from amacrine cells in the inner retina [4, 48]. Therefore, circadian Gα_z_ expression in photoreceptors may be driven by the clock within amacrine cells. Like *Gnaz*, the genes *Cpt-1α*, *Acadm* and *Nr4a1* have been identified to be under circadian and dopaminergic control in photoreceptors [49, 50]. Therefore, clock-dependent dopamine release from amacrine cells may be of general importance for driving rhythmicity of photoreceptor gene expression.

In contrast to D_4_ receptors, MT_1_ receptors do not play a role in driving rhythmicity of *Gnaz*. This follows from the present finding that notwithstanding the loss of functional MT_1_ receptors 24-h changes in *Gnaz* transcript are retained. However, the removal of functional MT_1_ receptors might result in advanced phasing of *Gnaz* (this study) and other genes shown to be rhythmic in photoreceptors including *Cpt-1α* and *Acadm* [50]. Therefore, melatonin acting on photoreceptor MT_1_ receptors [51, 52] might influence the phasing of gene expression rhythmicity in photoreceptors. Considering that melatonin is released by photoreceptors and may feedback on photoreceptor MT_1_ receptors, autocrine signaling might direct the phasing of gene rhythmicity in photoreceptors. Alternatively, melatonin might alter the phase of the clock in dopaminergic amacrine cells.

Daily rhythmicity of *Gnaz* is abolished in early diabetic retinopathy in the *db/db* mouse. Therefore, it might also be disturbed in diabetic retinopathy of humans—one of the most common causes of blindness in Europe and USA [53]. Since diabetic retinopathy affects dopamine content [54] and rhythmicity of *Drd4* expression (this study), the disturbed circadian regulation of *Gnaz* may reflect dysfunction of the retinal dopaminergic system under diabetic conditions. This assumption is consistent with the observation that circadian regulation of other genes under dopaminergic control is disturbed in diabetic retinopathy [50].

Circadian regulation of Gα_z_ suggests a role of Gα_z_ in linking the circadian clock to G protein-dependent signal transduction. Taken into account that Gα_z_ is known to regulate adenylyl cyclase activity [55, 56], it may contribute to circadian regulation of adenylyl cyclase activity and cAMP levels in retina [16, 57]. Since Gα_z_ is generally known to repress adenylyl cyclase activity, this assumption is consistent with the finding that the level of retinal Gα_z_—with a peak at late night and a nadir during day (this study)—is inversely correlated with the level of cAMP—showing a nadir at late night and a peak at dusk [16].

Gα_z_ has been conclusively linked to various types of GPCRs [58, 59]. Therefore, Gα_z_ might link the circadian clock to GPCR-dependent signal transduction. Interestingly, in tissues other than retina Gα_z_ has been shown to be coupled to D_2_-like receptors [60, 61]—a subclass of dopamine receptors that includes the dopamine D_4_ receptor type—and thus to a receptor family important for vision [17, 18, 62] and healthy retinal function [54]. However, data from immunoprecipitation studies (not shown) do not support direct coupling of Gα_z_ to dopamine D_4_ receptors in retina.

Interestingly, Gα_z_ has been seen in the SCN to be coupled to Gpr176, an orphan GPRC that sets the pace of circadian behavior [63]. This suggests a role of Gα_z_ in setting the phase of the master clock in the SCN. This assumption combined with rhythmicity of *Gnaz* in retina (this study) and pineal gland (this study) indicates that Gα_z_ is abundant and might play a clock-related role in all three components of the circadian system [43]. Accordingly, the α-transducin family appears to comprise clock- (*Gnaz*) and vision-related (*Gnat1*, *Gnat2*) G proteins.

In conclusion, the data of the present study suggest a concept in which Gα_z_ links the circadian clock and the dopaminergic system to GPCR signaling. Future investigations using Gα_z_ null mice [61, 64, 65] are warranted to reveal the exact function of Gα_z_ in photoreceptors and herewith its specific functional role in photoreceptor adaptation. The present data may also provide a suitable basis for future investigations dealing with the clock-related role of Gα_z_ in the circadian system.
